# A new species of leaf-toed gecko (Phyllodactylidae, *Phyllodactylus*) from María Cleofas Island, Nayarit, Mexico

**DOI:** 10.3897/zookeys.1024.60473

**Published:** 2021-03-15

**Authors:** Tonatiuh Ramírez-Reyes, Ilse K. Barraza-Soltero, Jose Rafael Nolasco-Luna, Oscar Flores-Villela, Armando H. Escobedo-Galván

**Affiliations:** 1 Posgrado en Ciencias Biológicas, Unidad de Posgrado, Edificio D, primer piso, Universidad Nacional Autónoma de México, Ciudad Universitaria 3000, Coyoacán, Ciudad de México, México; 2 Museo de Zoología, Facultad de Ciencias, Universidad Nacional Autónoma de México, Circuito Exterior de CU, Ciudad Universitaria, 04510, Ciudad de México, México; 3 Centro Universitario de la Costa, Universidad de Guadalajara, Av. Universidad 203, 48280, Puerto Vallarta, Jalisco, México; 4 Laboratorio de Ecología del Comportamiento, Centro de Investigaciones Biológicas del Noroeste, S.C., Av. Instituto Politécnico Nacional 195, Col. Playa Palo de Santa Rita Sur, Apdo. Postal 128; La Paz, BCS 23096, México; 5 Universidad Autónoma de Nayarit, Ciudad de la Cultura " Amado Nervo" s/n, 63155, Tepic, Nayarit, México

**Keywords:** Endemic gecko, genomics, insular species, morphological traits, Tres Marías Archipelago

## Abstract

We describe a new species of leaf-toed gecko of the genus *Phyllodactylus* from María Cleofas Island, the smallest island of Tres Marías Archipelago, Nayarit, México. Genomic, phylogenomic, and morphological evidence support that the new species presents a unique combination of diagnostic characters. Morphologically, the new species has a high number of tubercles, head to tail (mean 47), longitudinal ventral scales (mean 61), and third labial–snout scales (mean 26). Gene flow tests revealed the genetic isolation of insular populations from mainland counterparts. In addition, we confirmed the non-monophyly of *P. homolepidurus* and *P. nolascoensis*, and we show that the taxon *P. t. saxatilis* is a complex; therefore, we propose taxonomic changes within the *saxatilis* clade. The discovery of this new insular endemic species highlights the urgency of continued exploration of the biological diversity of island faunas of Mexico.

## Introduction

The family Phyllodactylidae currently includes 10 genera and 148 species of geckos (Uetz et al. 2020), with wide geographical distribution mainly in America, Africa, Europe, and the Middle East (Gamble et al. 2008; Vitt and Caldwell 2014). *Phyllodactylus* is among the 10 gecko genera with the highest number of species (Uetz et al. 2020). Within this diverse genus, particularly in Mexico, recent studies incorporating genomic data indicate a need for taxonomic changes and the description of new species that have remained undetected (or weakly differentiated) on the basis of morphological data alone (Ramírez-Reyes et al. 2020).

In the late nineteenth century, Stejneger (1899) reviewed the specimens of *Phyllodactylus* collected by E.W. Nelson and E.A. Goldman from the Islas Marías Archipelago (comprising the islands of María Madre, María Magdalena, María Cleofas, and San Juanito), and classified these populations as *Phyllodactylus tuberculosus*. Sixty years later, Zweifel (1960) collected additional individuals from María Madre Island and María Magdalena Island, and assigned these to the species *Phyllodactylus lanei* Smith, 1935. Pioneer efforts by James R. Dixon included an extensive taxonomic and systematic review of the genus *Phyllodactylus* in America (Dixon 1964, 1966, 1973; Dixon and Huey 1970). Based on morphological analysis, Dixon (1964) proposed the name *P. tuberculosus saxatilis* for gecko populations in Western Mexico, a wide geographic area including the states of Jalisco, Nayarit, Sinaloa, Sonora, Durango, Chihuahua and some islands off the coast of Nayarit. Dixon (1964) also mentioned some morphological differences between insular (from Islas Marías) and mainland populations of *P. t. saxatilis*, highlighting the morphological variation in the number of dorsal tubercles, tubercles between the axilla and groin, and the number of scales bordering the postmentals. These differences, however, were not statistically significant according to his criteria. Therefore, Dixon (1964) did not believe that this morphological variation justified a taxonomic distinction between insular and mainland populations. This taxonomic classification was subsequently accepted and published in checklists of the herpetofauna of Nayarit and the Isla Marías (McDiarmid et al. 1976; Casas-Andreu 1992; Nolasco-Luna et al. 2016; Woolrich-Piña et al. 2016).

Recently, Ramírez-Reyes et al. (2020) showed that the gecko population from María Cleofas Island was isolated and had differentiated from mainland lineages during the early Pliocene. Later, Ramírez-Reyes et al. (unpublished data) found that *Phyllodactylus tuberculosus* is a polyphyletic taxon and that none of the three subspecies studied (*P. t. tuberculosus*, *P. t. magnus* and *P. t. saxatilis*) share an exclusive more recent common ancestor with each other (i.e. share MRCA with other *Phyllodactylus*), and therefore, the evolution of each major clade occurred independently in time and space. In the *saxatilis* clade, the genetic differentiation of the María Cleofas Island lineage is particularly remarkable (Ramírez-Reyes et al. unpublished data). The gene flow or introgression test (ABBA-BABA) showed that this insular lineage has remained genetically isolated from mainland populations within the *saxatilis* clade. This last characteristic suggests treating this lineage as a candidate species under the biological species concept. However, there is uncertainty about the phylogenetic relationships of the insular lineage with respect to the continental lineages of the *saxatilis* clade. According to phylogenetic approximations (ML and Bayesian) based on the supermatrix (or supergene) of genomic data, the insular lineage of the *saxatilis* clade is related to *Phyllodactylus* lineages from Sinaloa (Ramírez-Reyes et al. 2020; Ramírez-Reyes et al. unpublished data). A species tree approach with coalescence methods could provide greater certainty about the phylogenetic relationships of lineages and species within the *saxatilis* clade. Given the set of evidence that allows defining this new species, the main objective of this study is the formal description of the insular lineage from María Cleofas Island, which adds an endemic taxon to the herpetofauna of the Tres Marías Archipelago. In addition, we elaborate a coalescence-based species tree for the *saxatilis* clade from SNP data, and assess the taxonomic treatment of the lineages and candidate species within the *saxatilis* clade.

## Materials and methods

### Study area

**Figure 1.**
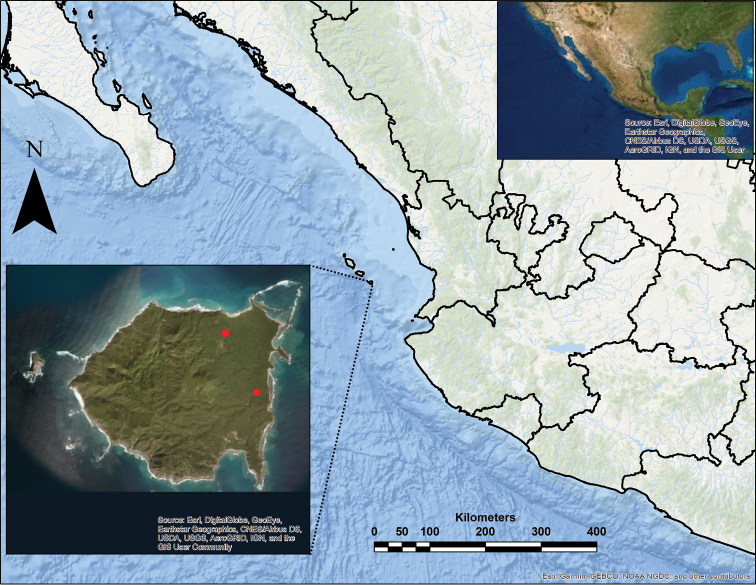
Map of Mexico showing specimen collection sites on Maria Cleofas Island (red dots).

**Figure 2.**
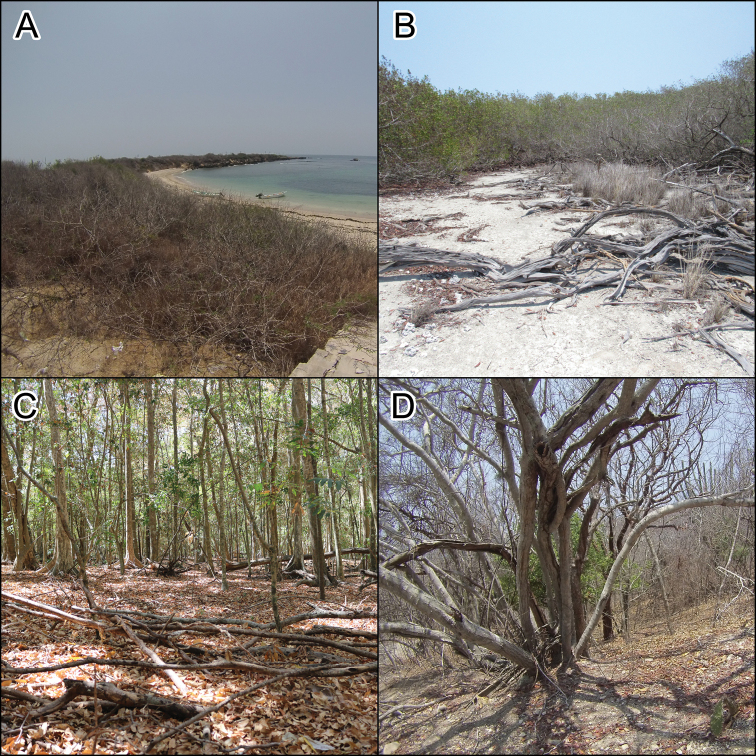
Vegetation typed on María Cleofas Island **A** scrub forest **B** small area of *Rhizophora mangle***C** tropical sub-deciduous, and **D** deciduous forest.

María Cleofas Island was well described by Emerson (1958), Zweifel (1960), and Casas-Andreu (1992). Of the four islands that comprise the Islas Marías Archipelago Biosphere Reserve, María Cleofas Island is the closest to the mainland, at a distance of 87.5 km to the nearest continental point located in San Blas, Nayarit, and has a surface area of 25 km^2^ (Fig. 1). Tropical sub-deciduous and deciduous forests, scrub forests, and a small area of *Rhizophora mangle* dominate the vegetation (Fig. 2). The climatic conditions are characterized by a dry season from November to April and a rainy season from May to October (Bullock 1986). The island shows an average ambient temperature of 24.9 °C; the minimum and maximum temperatures are 21.1 °C and 28.7 °C in January–February and July, respectively. The mean annual rainfall is 564.2 mm, of which 95% occurs from June to December (CONANP 2007).

### Sampling

**Figure 3.**
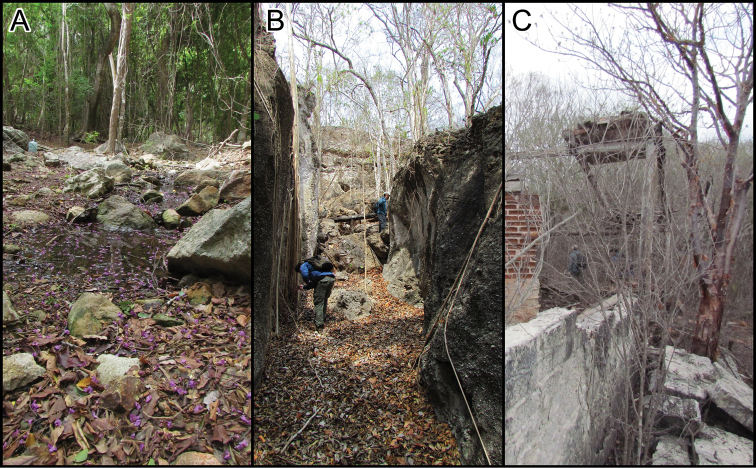
Habitat types where geckos were captured during diurnal and nocturnal surveys on María Cleofas Island. Most individuals were observed/captured on isolated rocks **A** or rock-walls **B** along a dried streambed. Some individuals were captured on the remains of human-fabricated construction.

We performed diurnal and nocturnal surveys in all available habitats to assess the herpetofauna of the María Cleofas Island from May 2017 to August 2018. The survey consisted of 2–3 researchers walking *ad libitum* through the different types of vegetation on the island, examining every microhabitat that could be potentially utilized by amphibians and reptiles (Fig. 3). Our field observations were conducted during the day from 09:00 to 17:00 h, and at night from 21:00 to 04:00 h. All captured lizards were taken to the María Cleofas Research Boat for sampling and data collection (see below for morphological analyses). We captured 36 individuals of the genus *Phyllodactylus* during fieldwork under collecting permits SGPA/DGVS/01208/17 and SGPA/DGVS/010144/18, of which tissue samples of two specimens were used for molecular analyses (Ramírez-Reyes et al. 2020), and 11 specimens were used for the present morphological description. We only sacrificed nine individuals (the type series) by injection of sodium pentobarbital, following the animal ethics guidelines of UNAM.

### Genomic data and species tree

We used the generated variant calling format file (VCF) of Ramírez-Reyes et al. (unpublished data), which contains the genetic variants (SNPs) corresponding to the *saxatilis* clade. This VCF file contains 13 genotypes and 14,271 binary SNPs (8.1% missing data). We analyzed this VCF file in R, mainly using three packages: vcfR (Knaus and Grünwald 2017), adegenet (Jombart and Ahmed 2011), and StAMPP (Pembleton et al. 2013). With these packages, we calculated Nei’s distances between populations, as well as pairwise F_ST_ among populations. We visualized Nei’s distances as a Neighbor joining (NJ) tree obtained in Rstudio.

We elaborated a species tree in SVDquartets (Chifman and Kubatko 2014, 2015) implemented in PAUP* v. 4 (Swofford 2003) with the 14,271 binary SNPs. The individual samples (n = 13) from the VCF file were assigned to seven species, based upon previous studies about evolution and candidate species in the *saxatilis* clade (Ramírez-Reyes et al. 2020; Ramírez-Reyes et al. unpublished data). We performed a species tree search using exhaustive quartet sampling (289 quartets) and used the QFM assembly algorithm to assemble the species tree. We also indicated the multispecies coalescent tree model. Branch support was assessed using 100 nonparametric bootstrap replicates.

### Morphological analyses

We collected meristic character counts (n = 104) and morphometric measurements (n = 117) of specimens with an electric digital caliper (resolution 0.00005"/0.01 mm). We examined 117 specimens from four scientific collections: Colección Nacional de Anfibios y Reptiles at Instituto de Biología (CNAR-IBUNAM), Museo de Zoología de la Facultad de Ciencias at Universidad Nacional Autónoma de México (MZFC-UNAM), Colección de Anfibios y Reptiles at Escuela Nacional de Ciencias Biológicas (ENCB-IPN), and the Royal Ontario Museum of Canada (ROM). Nine individuals from María Cleofas Island were deposited at MZFC-UNAM with collection numbers MZFC-HE 35618–35626 (see the description).

All measurements and counts followed the protocol of Ramírez-Reyes and Flores-Villela (2018). Measured characters were: **SVL** snout-vent length, **AG** axilla-groin length, **HW** head width, **SE** snout-eye length, **ED** eye diameter, **AO** auricular opening, **IOD** interorbital distance, **IND** internarial distance, **LFF** length of the fourth finger, and **LFT** length of the fourth toe. The meristic characters counted were: **DLF** digital lamellae of the fourth finger and toe (left and right, four total counts), **THT** tubercles from head to tail, **TAG** tubercles from axilla to groin, **RTD** rows of tubercles across the dorsum, **IS** interorbital scales, **SBI** scales bordering internasals, **TSS** third labial–snout scales, **SEN** scales between nostril and eye, **SBP** scales bordering postmentals, **SAV** scales across venter, and **LVS** longitudinal ventral scales.

We conducted multivariate analyses for morphological characters using PCA and MANOVA to determine significant morphological differences among species within the *saxatilis* clade. A one-way ANOVA was performed to compare the mean SVL of all species analyzed, as well as the differentiated characters suggested by Dixon (1964): number of dorsal tubercles, tubercles between axilla and groin, and number of scales bordering the postmentals. Finally, the meristic characters were analyzed by multivariate analysis (MANOVA). All statistical analyses (morphometric and meristic characters) were performed in Rstudio.

### Diet

We analyzed the stomach content from 36 individuals during fieldwork using stomach flushing (Legler and Sullivan 1979). In contrast with dissection techniques that require sacrificing the animals to obtain stomach contents, the aforementioned technique enables individuals to regurgitate prey items for further analysis (Perkins and Eason 2019). We performed this technique three times consecutively to obtain the largest possible volume of stomach contents from each individual (Barraza-Soltero 2019; Barraza-Soltero and Escobedo-Galván 2020). The captured geckos were taken to the María Cleofas Research Boat for sampling and data collection. After data collection, geckos were under observations between 24 to 36-hours before being returned to the capture sites on the island. During this time, we observed no deaths due to manipulation. Stomach contents were preserved in 70% ethanol for laboratory analysis at the Biodiversity and Ecosystem Services Laboratory of the Universidad de Guadalajara, Puerto Vallarta, Jalisco, Mexico. The stomach contents were separated using a Carl Zeiss Stemi DV4 stereoscopic microscope and were examined under an Olympus optical microscope. Prey items were identified to the lowest taxonomic level possible using specialized literature. In addition, confirmation and identification of some prey species was done by Fabio G. Cupul-Magaña (CUC, Universidad de Guadalajara, México).

## Results

### Genomic evidence and species tree

**Figure 4.**
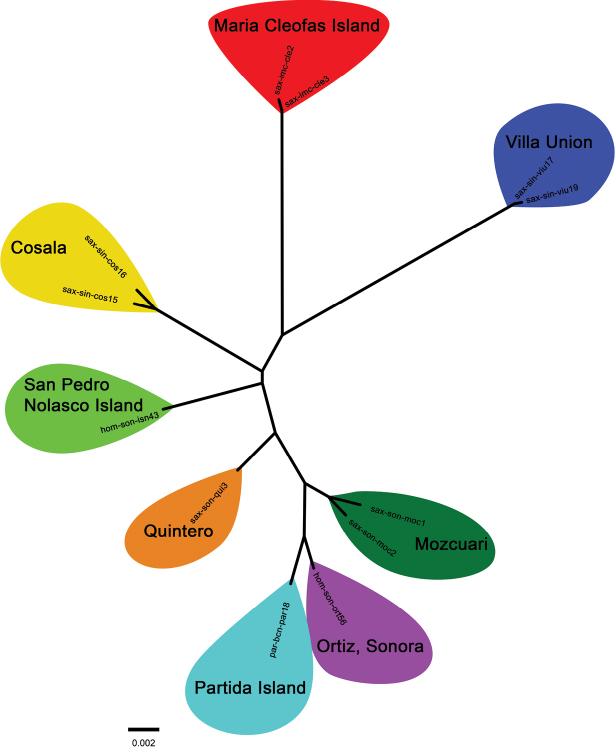
Neighbor-joining (NJ) tree based on Nei’s genetic distance for five populations of *P. t. saxatilis* (Maria Cleofas Island, Cosala, Villa Union, Quintero, and Mocuzari), two populations of *P. homolepidurus* (San Pedro Nolasco Island and Ortiz) and *P. partidus* (Partida Island) from Mexico (island and mainland populations).

The F_ST_ values supported a very high genetic differentiation among the populations studied (F_ST_ = 0.82 on average among all populations; Table 1). In particular, for *P. t. saxatilis* from the María Cleofas Island population (the focal population in this study), F_ST_ was 0.92, on average, with respect to all other populations. The lowest F_ST_ value (0.36) was calculated between the populations of *P. t. saxatilis* from Mocuzari and *P. homolepidurus* (mainland). Nei’s distance values were consistent with F_ST_ values; we obtained a distance of 5% on average among all populations, based on SNP data. These differences among individuals and populations were clearly supported and illustrated in the NJ tree (Fig. 4). The NJ topology showed that the most differentiated populations of the *saxatilis* clade were those from María Cleofas Island and Villa Union.

**Table 1.** Nei’s distances and pairwise F_ST_ below and above the diagonal, respectively, among the studied populations calculated from the SNP matrix. The Maria Cleofas Island (MCI) lineage is shown in bold.

	*P. delcampi*	*P. nolascoensis*	*P. homolepidurus*	*P. partidus*	*P. t. saxatilis* (MCI)	*P. t. saxatilis* (C)	*P. t. saxatilis* (VU)	*P. t. saxatilis* (M)	*P. t. saxatilis* (Q)
*P. delcampi*	–	NC	NC	NC	**0.99**	0.96	0.98	0.95	NC
*P. nolascoensis*	0.18	–	NC	NC	**0.95**	0.71	0.93	0.66	NC
*P. homolepidurus*	0.18	0.02	–	NC	**0.93**	0.79	0.92	0.36	NC
*P. partidus*	0.19	0.02	0.01	–	**0.96**	0.8	0.95	0.48	NC
***P. t. saxatilis* (MCI)**	**0.18**	**0.02**	**0.03**	**0.03**	–	**0.86**	**0.94**	**0.83**	**0.91**
*P. t. saxatilis* (C)	0.18	0.02	0.02	0.02	**0.03**	–	0.86	0.72	0.69
*P. t. saxatilis* (VU)	0.19	0.03	0.04	0.03	**0.03**	0.03	–	0.85	0.9
*P. t. saxatilis* (M)	0.18	0.02	0.01	0.01	**0.03**	0.02	0.03	–	0.43
*P. t. saxatilis* (Q)	0.18	0.02	0.01	0.01	**0.02**	0.02	0.03	0.01	–

C = Cosala, VU = Villa Union, M = Mocuzari, Q = El Quintero, NC = not calculated.

**Figure 5.**
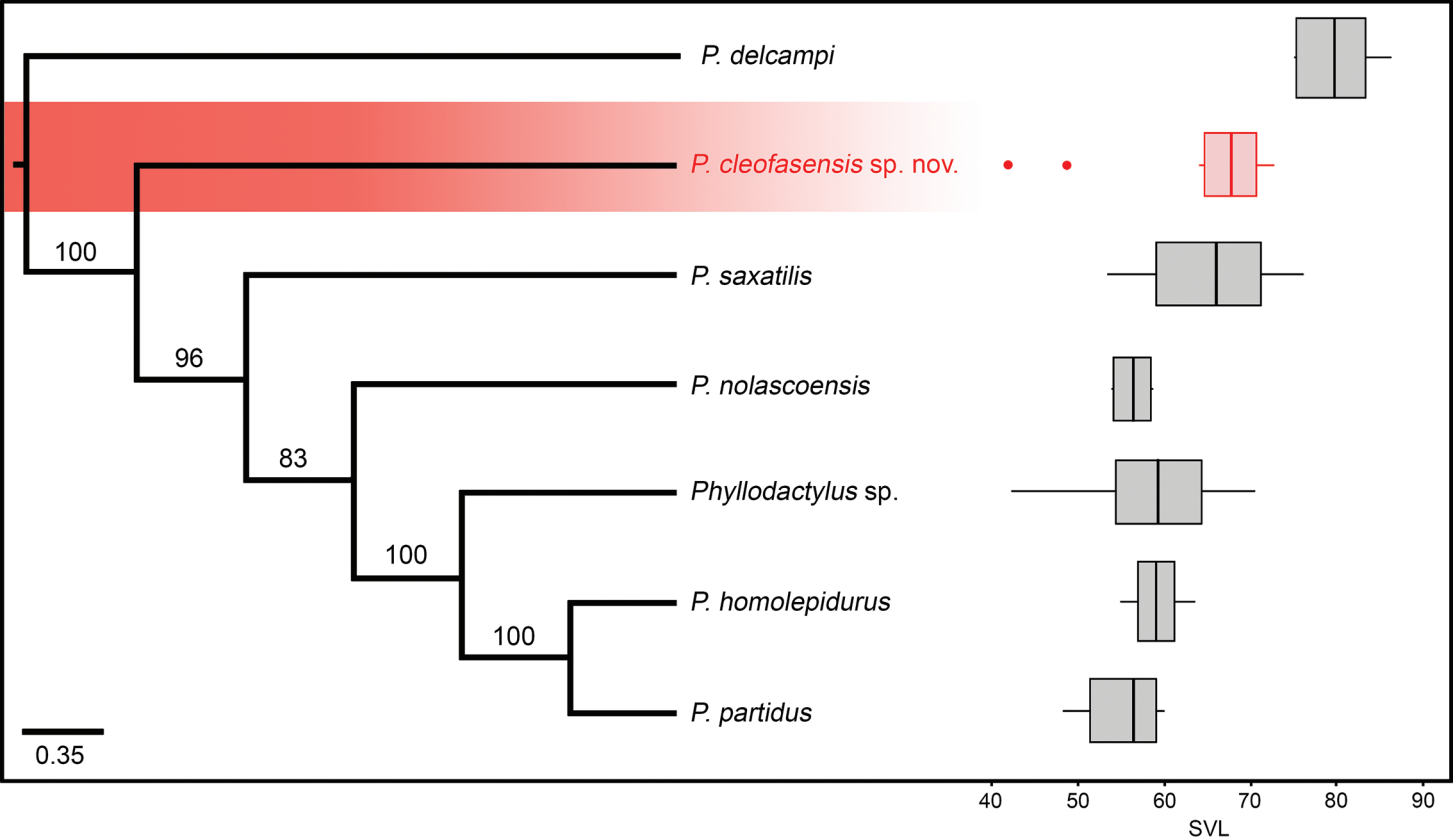
Species tree inferred with SVDquartets. Bootstrap support values are indicated above the branches (significant bootstrap values > 75). In addition, boxplot of SVL for each species with outliers shown with dots.

The species tree developed in SVDquartets showed a high percentage of compatible quartets (87.5%) versus incompatible quartets (12.5%). The recovered species tree shows that the mainland populations of *P. t. saxatilis* (North and South) are rendered non-monophyletic by *P. nolascoensis*, *P. homolepidurus*, and *P. partidus*. This result is broadly consistent with previous studies, in which the non-monophyly of *P. t. saxatilis* was demonstrated (Ramírez-Reyes et al. 2020; Ramírez-Reyes et al. unpublished data). This fundamental difference between populations in southern Sinaloa with respect to those in Sonora (both previously considered as *P. t. saxatilis*) requires the delimitation of *P. saxatilis* in order to avoid non-monophyletic groups. We consider that *P. saxatilis* be restricted to the populations from the type locality (Villa Union, Sinaloa) and surrounding areas (Dixon 1964). Thus, *P. cleofasensis* sp. nov. (see description) is related to *P. saxatilis*, while the other insular species *P. nolascoensis* is related to *Phyllodactylus* sp. and the two sister species (*P. homolepidurus* + *P. partidus*) (Fig. 5).

### Morphological analyses

The results of the meristic counts and morphological measurements are summarized in Tables 2 and 3, respectively. All statistical analyses are based on adult individuals. The ANOVA results for the four characters analyzed rejected the null hypothesis of equality of means among the six compared species with high significance (Table 4). Of the four characters analyzed, SVL obtained the highest significance and was supported by Tukey’s paired test, of which we highlight the differences between *P. magnus*-*P. cleofasensis* sp. nov. (P = 0.009), *P. nolascoensis*-*P. magnus* (P < 0.001), *P. partidus*-*P. magnus* (P < 0.001), *Phyllodactylus* sp. (Son)-*P. magnus* (P < 0.001), and *P. saxatilis*-*P. partidus* (P = 0.089). Regarding RTD, Tukey tests showed a greater difference between *Phyllodactylus* sp. (Son) – *P. cleofasensis* sp. nov. (P = 0.01). The AGT Tukey tests supported the differences between *P. partidus*-*P. cleofasensis* sp. nov. (P = 0.01), *P. saxatilis*-*P. cleofasensis* sp. nov. (P = 0.002), *Phyllodactylus* sp. (Son)-*P. cleofasensis* sp. nov. (P = 0.01), *P. partidus*-*P. nolascoensis* (P = 0.05), and *P. saxatilis*-*P. nolascoensis* (P = 0.02). Finally, for SBP Tukey tests showed significant differences between *P. partidus*-*P. magnus* (P = 0.006) and *Phyllodactylus* sp. (Son)-*P. magnus* (P = 0.01).

**Table 2.** Mean and (standard deviation) of meristic characters examined for species studied. In bold we show the set of diagnostic meristic characters of *P. cleofasensis* sp. nov.

Species	L4AR	L4AL	L4PR	L4PL	THT	TAG	RTD	IS	SBI	TSS	SEN	SBP	SAV	SLV
*P. cleofasensis* sp. nov.	11.71 (1.49)	12.63 (0.51)	13.25 (1.03)	13.67 (1.22)	**47.89 (7.3)**	24.78 (4.86)	15 (0.75)	21.44 (2.40)	7 (0)	**26.55 (2.35)**	12.55 (0.52)	7.33 (0.5)	27.22 (3.8)	**61 (4.33)**
*Phyllodactylus* sp. (Sonora)	11.56 (1.04)	11.6 (0.80)	12.63 (1.09)	12.70 (0.90)	**31.36 (4.51)**	20.48 (2.71)	13.24 (1.25)	21.96 (2.59)	6.78 (0.69)	**24.30 (1.77)**	11.18 (0.95)	7.48 (0.97)	26.7 (3.48)	**58.03 (3.84)**
*P. saxatilis*	10.87 (0.64)	11.25 (1.03)	12.25 (1.03)	11.62 (0.74)	**32.32 (7.6)**	18.25 (3.99)	13.62 (1.76)	20.12 (2.53)	6.5 (0.83)	**23.87 (1.95)**	11.75 (0.70)	6.8 (0.64)	26.37 (3.29)	**55 (3.96)**
*P. nolascoensis*	10.5 (0.7)	11 (1.41)	12 (0.81)	13 (1.15)	**41.75 (1.5)**	25 (3.55)	15.25 (2.06)	23 (3.26)	7 (0)	**24.75 (1.5)**	12 (1.15)	8 (0.81)	24.75 (3.4)	**52.75 (1.89)**
*P. magnus*	11.53 (1.14)	11.75 (1.03)	12.32 (1.14)	11.95 (1.28)	**34.82 (5.80)**	21.90 (3.75)	13.64 (1.47)	22.46 (2.72)	6.86 (0.53)	**25.59 (1.86)**	11.78 (0.90)	6.73 (1.13)	25.19 (2.13)	**55.30 (4.67)**
*P. partidus*	10.8 (0.44)	11 (0.70)	11.8 (1.3)	12 (0.81)	**33 (2.94)**	18.2 (3.11)	13 (0.70)	19.4 (2.07)	6 (0.70)	**23.8 (2.38)**	11.6 (1.14)	8.4 (0.54)	27.2 (1.92)	**55.2 (2.77)**

**Table 3.** Mean (and standard deviation) of morphometric characters (in millimeters) for the studied species.

Species	SVL	AG	HW	SE	ED	AO	IOD	IND	LFF	LFT
*P. cleofasensis* sp. nov.	64.19 (10.38)	25.42 (3.78)	12.53 (2.45)	9.37 (2.68)	4.29 (0.45)	2.53 (0.19)	8.0 (1.61)	2.12 (0.48)	5.49 (0.98)	6.37 (0.99)
*P. saxatilis*	65.44 (7.77)	26.89 (2.61)	12.23 (1.44)	10.62 (1.14)	3.97 (0.35)	2.09 (0.37)	8.20 (1.24)	2.23 (0.33)	4.82 (0.62)	6.33 (1.08)
*Phyllodactylus* sp. (Sonora)	58.85 (7.36)	23.01 (3.74)	10.90 (1.45)	9.25 (1.04)	3.62 (0.45)	1.82 (0.33)	7.12 (0.89)	1.83 (0.28)	4.63 (0.77)	5.76 (0.77)
*P. nolascoensis*	56.29 (2.54)	23.85 (4.14)	10.38 (0.35)	8.95 (0.35)	3.94 (0.16)	2.17 (0.13)	6.56 (0.13)	1.9 (0.13)	4.73 (0.35)	5.15 (0.51)
*P. magnus*	73.03 (6.85)	29.61 (3.33)	13.51 (1.44)	12.04 (1.14)	4.68 (0.51)	2.57 (0.47)	8.85 (0.85)	2.42 (0.36)	5.72 (0.73)	6.94 (0.92)
*P. partidus*	55.12 (4.89)	20.71 (2.4)	10.67 (1.29)	9.19 (1.03)	3.55 (0.46)	1.97 (0.42)	7.06 (0.85)	2.08 (0.32)	4.67 (0.50)	5.49 (0.53)

**Table 4.** Summary of ANOVA tests for the morphometric character snout-vent length (SVL) and three meristic character: row dorsal tubercles (RDT), axilla-groin tubercles (AGT), and scales bordering postmentals (SBP).

Character	Df	Sum. Sq.	Mean sq.	F value	Pr (>F)
SVL	5	5472	1094.4	20.38	2.38 × 10^-14^***
RDT	5	34.35	6.87	3.71	0.00406**
AGT	5	324.4	64.87	5.326	0.000226***
SBP	5	23.1	4.621	4.761	0.000616***

** P < 0.05; *** P < 0.001.

**Figure 6.**
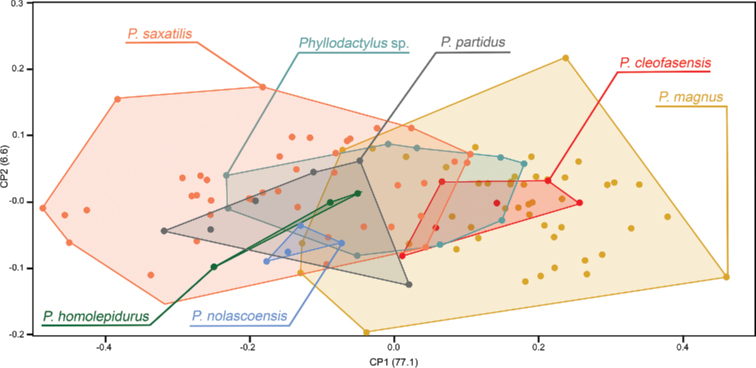
Scatter plot of nine morphometric variables for seven species of the *P. t. saxatilis* clade. The first two components are shown with the percentage of variance on the axes.

The PCA of morphometric data (excluding SVL) for the six species studied (Fig. 6) showed that about 83% of the variance was explained by the first three components. However, plotting individuals by the main components does not recover any group independently; furthermore, it shows that high overlap exists in the morphometric characters analyzed (Fig. 6). However, as mentioned previously, SVL is the most informative morphometric character; we can even visualize the differences in size of the studied species. For the *saxatilis* clade, *P. cleofasensis* sp. nov. and *P. saxatilis* have the largest body size, while the insular *P. partidus* and *P. nolascoensis* are the smallest (Fig. 5).

For the meristic characters, we performed the MANOVA excluding the SBI, TSS, and SEN characters since they did not show a normal distribution (or close to it). All MANOVA tests rejected the equality of multivariate means for all species analyzed (P < 0.001), Pillai trace (Trace = 1.51, d.f. = 5, F = 3.6, P = 1.73 × 10^-14^), Wilk's lambda (λ = 0.14, F = 3.9, P = 5.715 × 10^-16^), Hotteling test (HL = 2.66, F = 4.2, P = 2.2 × 10^-16^) and Roy test (R = 1.26, F = 10.5, P = 2.99 × 10^-12^). Once we determined that the multivariate means were not the same, we proceeded to perform individual ANOVA on paired comparisons of the most significant characters (P < 0.001), but emphasizing the differences of *P. cleofasensis* sp. nov. with respect to the others. For example, in the case of THT, *P. cleofasensis* sp. nov. differs significantly with respect to all species (P < 0.001) except *P. nolascoensis*, a pattern also seen with TAG and RTD characters. On the other hand, the ventral longitudinal scales demonstrated a significant difference between *P. cleofasensis* sp. nov. and *P. magnus* (P < 0.001), *P. nolascoensis* (P < 0.001), *P. partidus* (P = 0.009) and *P. saxatilis* (P = 0.002), while the lowest significance value for this character was with *Phyllodactylus* sp. (P = 0.04).

### Taxonomy

The present study constitutes a precedent for the taxonomy of the *saxatilis* clade (*sensu* Ramírez-Reyes et al. unpublished data) based on analyses of genomic and phylogenomic data. Here, our taxonomic treatment allows the use of specific categories for the studied populations and avoids non-monophyletic groups and infraspecific categories (*P. t. saxatilis*, *P. homolepidurus*). Specifically, in the case of the endemic population from the María Cleofas Island, the genomic evidence suggests that this population is clearly differentiated from its mainland counterparts (Ramírez-Reyes et al. 2020; Ramírez-Reyes et al. unpublished data), and a formal description of this species is warranted.

#### Species description

##### Phyllodactylus cleofasensis sp. nov.

Taxon classificationAnimaliaSquamataPhyllodactylidae

E0BD74DF-8214-5003-A5B6-1763858FD1E1

http://zoobank.org/0FCA70B9-5FF9-4AE4-B422-7210A3F1ED4F

Fig. 7

Phyllodactylus tuberculosus (in part) Wiegmann 1835 (Stejneger 1899)
Phyllodactylus lanei (in part) Smith 1935 (Zweifel 1960)
P. tuberculosus saxatilis (in part) Dixon 1964 (McDiarmid et al. 1976; Casas-Andreu 1992; Woolrich-Piña et al. 2016)

###### Common name.

María Cleofas leaf-toed Gecko, Salamanquesa de la Isla María Cleofas.

###### Type species.

***Holotype*:** Adult female (MZFC-HE 35623) collected on María Cleofas Island (21.3095°N, 106.2340°W, WGS84, 64 m elev.) on 24–25 May 2018 by Ilse K. Barraza Soltero and Armando H. Escobedo Galván. ***Paratypes*.** All collected from the type locality, María Cleofas Island (Six adult specimens and two juveniles). MZFC-HE 35618-35622 and MZFC-HE 35624-35626.

**Figure 7.**
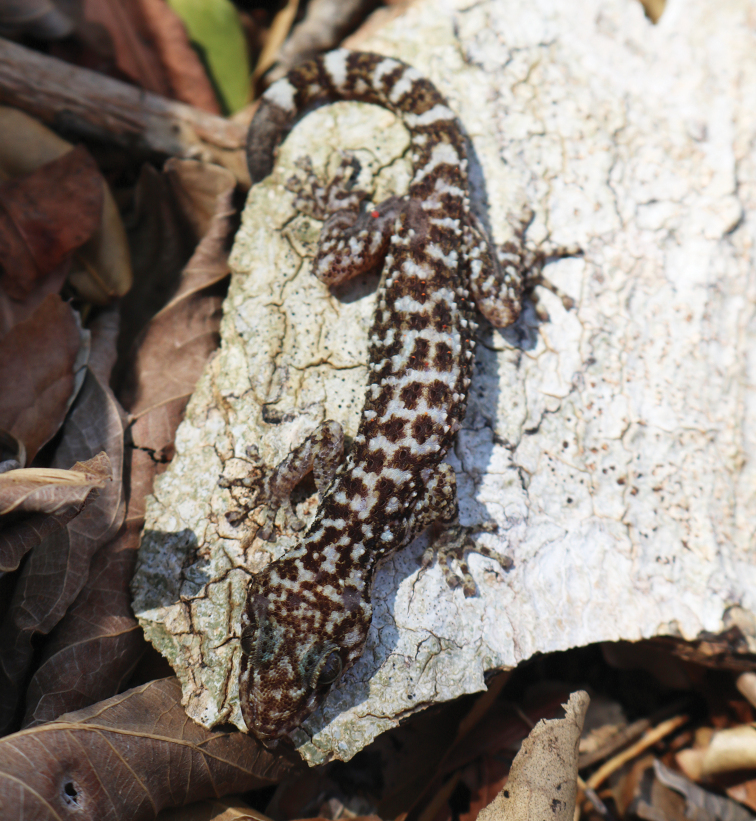
Dorsal view of *Phyllodactylus cleofasensis* sp. nov., in life. (Photo by AHE-G).

###### Diagnosis.

*Phyllodactylus cleofasensis* is a species of medium to large body size. Snout-vent length of *P. cleofasensis* measured during fieldwork ranged from 44.8 to 77.0 mm (mean 59.5 mm). Concerning body size, *P. cleofasensis* differs (in mean size) from *P. magnus* (73.03), *P. nolascoensis* (56.29), *P. partidus* (55.12) and *P. saxatilis* (65.44). *Phyllodactylus cleofasensis* has a white venter, variable dorsal coloration, and a greater number of paravertebral dorsal tubercles (mean 47.89). Three meristic characters mainly differentiate *P. cleofasensis* from the rest of the species of the *saxatilis* clade (*P. saxatilis*, *P. nolascoensis*, *P. partidus*, *P. homolepidurus* and *Phyllodactylus* sp.) (Table 3): paravertebral dorsal tubercles from head to tail (mean 47.89); number of scales across the snout, starting from the 3^rd^ labial scale (mean 26.5); and number of longitudinal ventral scales from an imaginary line of the forelimbs to the cloacal opening (mean 61). *Phyllodactylus cleofasensis* has the highest values of the mentioned characters, while the rest of the studied species presented lower counts compared to other *Phyllodactylus* species in Mexico (Tables 2 and 3). The new species has the second highest mean number of dorsal tubercles after *P. delcampi* (63.4). Other *Phyllodactylus* species have fewer mean dorsal tubercles (*P. paucituberculatus*, 28.7; *P. duellmani*, 37.1; *P. bordai*, 32.9; *P. davisi*, 41.6; *P. muralis*, 33.1; *P. homolepidurus*, 35.9; *P. xanti*, 37; *P. lanei*, 32.4; *P. papenfussi*, 33.2; *P. isabelae*, 32.3; *P. lupitae*, 28.8; *P. rupinus*, 28; *P. benedettii*, 28.8; and *P. kropotkini*, 28.4). Regarding the number of scales crossing the snout, *P. cleofasensis* has a very similar number of scales to *P. muralis* (26.1) and *P. lupitae* (25.5); lower counts occur in *P. unctus* (21.7), *P. paucituberculatus* (19.5), *P. duellmani* (19.9), *P. delcampi* (21), *P. bordai* (19.4), *P. davisi* (23.7), *P. muralis* (26.1), *P. homolepidurus* (22.5), *P. xanti* (16.5), *P. lanei* (21.6), *P. papenfussi* (17.8), *P. isabelae* (21.1), *P. rupinus* (20.6), *P. benedettii* (22), and *P. kropotkini* (20.2). Concerning ventral scales, *P. cleofasensis* has a mean of 61, similar to three species of the *lanei* clade (or clade I), namely, *P. lupitae* (61.5), *P. rupinus* (62) and *P. lanei* (62.86). The rest of the *Phyllodactylus* in Mexico have less than 61 scales: *P. kropotkini* (60), *P. benedettii* (59.71), *P. xanti* (58.75), *P. angelensis* (56.75), *P. davisi* (55.5), *P. magnus* (55.3), *P. partidus* (55.2), *P. saxatilis* (55), *P. santacruzensis* (54), *P. muralis* (53.9), *P. isabelae* (53.85), *P. nolascoensis* (52.75), *P. homolepidurus* (52.5), *P. bordai* (51.91), *P. bugastrolepis* (51.57), *P. duellmani* (48.75), *P. paucituberculatus* (48.66), and *P. unctus* (48.5).

###### Description of the holotype.

Adult female with SVL 71.17 mm, robust body, head not flattened, neck slightly differentiated from head. Head width 14.4 mm and snout length 12.11 mm. Rostral scale is flat (no grooves or stretch marks) and in contact with the two internasal scales. Nostril in contact mostly with the rostral scale and marginally with the first labial scale on both sides; supranasal scales in contact with 12 scales crossing from right to left side; 22 interorbital scales counted from middle of eye, interorbital distance 9.75 mm. 24 scales crossing snout between contralateral second labial scales 27 between third labial scales. Number of loreal scales 14 on right side and 15 on left side; equal number of supralabial scales (14), labial scales (8), and infralabial scales (7) on each side. Auricular opening oval (2.48 mm) smaller than the ocular opening, occipital scales similar in size and shape to interorbitals (not greatly differentiated in size and shape). Mental scale slightly wider than long, forming a “V” but not with pronounced angles; eight postmental scales in contact with first infralabials on both sides (right and left). Body with granular and circular scales interspersed with tubercles of different sizes; the specimen presents a fragmented tail. Internarinal distance 2.67 mm and axilla-groin length 26.19 mm. 14 rows of dorsal tubercles, 20 axilla-groin tubercles on right side and 19 on left side. Presents 29 transversal ventral scales with first ventral scale differentiated from lateral scales (which are small and circular). Ventral scales differentiated in size and shape from lateral and gular scales. Scales imbricate on extremities (anterior and posterior), as well as on dorsal region of the tail. No femoral or precloacal pores. Digital lamella formulae: right posterior (9-11-15-14-13), left posterior (8-10-11-12-13), right anterior (8-11-12-13-10), left anterior (8-10-12-13-11); fourth finger of extremities longer that others (5.55 mm manus, 7.49 mm pes); digital toepads longer than wide on all fingers.

###### Etymology.

Specific epithet is taken from the type locality María Cleofas Island, with the Latin suffix -*ensis* meaning, “originating from.” Specific epithet is masculine, in agreement with the gender of *Phyllodactylus*.

###### Variation.

All meristic and morphometric characters are presented with mean values and standard deviation in Tables 2 and 3, respectively.

###### Natural history.

**Figure 8.**
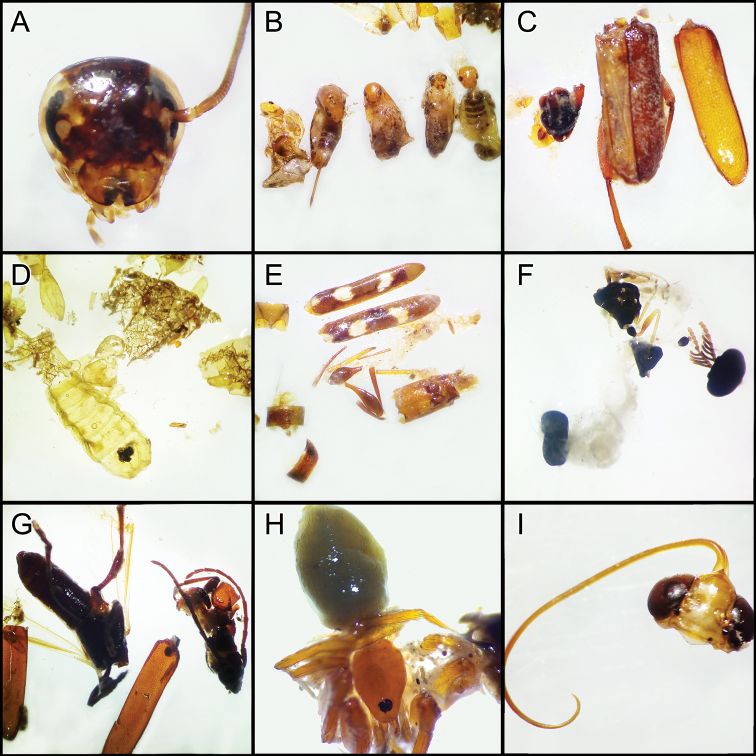
Prey items identified from stomach contents of *P. cleofasensis***A***Pycnoselus surinamensis***B** crickets of the family Rhaphidophoridae**C** beetles of the genus *Telabis***D***Centruroides elegans insularis***E***Euphoria germinata***F** and **G** beetles of the family Elateridae**H** spider of the family Caponiidae**I** some individuals of lepidopterans.

Individuals of *P. cleofasensis* were observed active during night surveys under single rocks, abandoned anthropogenic structures, or in some cases on the trunk of *Piranhea mexicana*. Some were observed on the ground while they moved between rocks. In some parts of the island, they can be seen in abandoned man-made structures, which are shelters for species such as geckos, anoles, iguanids, and bats. Although predation of *P. cleofasensis* has not been reported, we suggest that *Oxybelis microphthalmus* (for taxonomic status see Jadin et al. 2020) could be a potential predator given that we captured two *O. microphthalmus* at the same site of *P. cleofasensis* during nocturnal surveys. During our visits (May to August 2017 and April to August 2018), we did not observe reproductive activity or females with a shelled egg in one of their oviducts. Eleven distinct prey items were found in 36 stomach contents. It has been mentioned that the diets of lizards in island environments contain a high percentage of vegetation matter due to the lack of food or scarcity of prey belonging to the Class Insecta (Olesen and Valido 2003). However, in this work, a great variety of arthropods was reported in the stomach contents of geckos (Fig. 8). Prey items of *P. cleofasensis* were mostly composed of orthopterans (Family Rhaphidophoridae) and coleopterans (Family Passalidae); they also consumed plant matter, arachnids, lepidopterans, scorpions (*Centruroides elegans insularis*), and cockroaches (Blattodea: *Pycnoselus surinamensis*) (Fig. 8). Additionally, during fieldwork in May 2018, we captured two individuals with remains of shed skin in their stomach contents (Barraza-Soltero and Escobedo-Galván 2020).

## Discussion

Herein we present genomic, phylogenomic, and morphological evidence that demonstrates the independent species status of the endemic population of *Phyllodactylus* from María Cleofas Island. To our knowledge, the only work that mentioned morphological differences between the insular and mainland populations of *P. t. saxatilis* was conducted by Dixon (1964). Fifty-six years later, we confirmed that these differences were supported by genetic isolation of this population from those on the mainland using genomic data (Ramírez-Reyes et al. 2020; Ramírez-Reyes et al. unpublished data). Surprisingly, *Phyllodactylus cleofasensis* is the third gecko species to be recognized from the islands located off the coast of Nayarit, together with *P. isabelae* endemic to the Marietas Islands and *P. lupitae* endemic to El Coral Island (Ramírez-Reyes and Flores-Villela 2018). In the 1960’s, there was a consensus among herpetologists that the insular amphibians and reptiles from the Western Mexican Pacific were representatives of taxa found on the adjacent mainland, due to a possible Neotropical influence in their evolution (Casas-Andreu 1992). However, recent molecular techniques have challenged the traditional view of the herpetofauna on both the mainland and the islands of the Central Pacific of Mexico. For example, Card et al. (2016) recently recognized independent lineages of the genus *Boa* for the Mexican Pacific coast. Similarly, Ramírez-Reyes et al. (2017) analyzed and confirmed divergent lineages within the *Phyllodactylus lanei* complex. In light of this diversity, increased research efforts in alpha taxonomy should be considered in conjunction with the generation of genetic and genomic resources of insular fauna. The scarcity of current studies reflects minimal interest in the conservation of the insular diversity of Mexico, and at the same time maintains the great uncertainty of the *status quo* of the herpetofaunistic diversity of insular ecosystems (Blázquez et al. 2018).

The phylogenetic position of *P. cleofasensis* was unknown until recently. Based on a relaxed molecular clock model, this species diverged from mainland populations during the late Miocene (~7 mya), and shows the highest genetic isolation of the species in the *saxatilis* clade (Ramírez-Reyes et al. unpublished data). The recovered species tree allows us to propose a more stable taxonomy for this clade, with a clear separation of *P. homolepidurus* and *P. nolascoensis* (as was recently proposed by Lemos-Espinal et al. 2019), and some non-monophyletic lineages that were considered part of *P. t. saxatilis* (*Phyllodactylus* sp. in this study). Only a broader sampling will establish if there are significant morphological differences between *P. homolepidurus* and *P. nolascoensis*, as mentioned by Dixon (1964). Meanwhile, our taxonomic treatment allows the use of specific categories for the studied populations and avoids polyphyletic groups and infraspecific categories (*P. saxatilis* and *P. homolepidurus*). Similar to this study, Koch et al. (2016) implemented an integrative taxonomic framework to propose an updated and stable taxonomy for the *P. reissi* complex in Perú.

The three insular endemic species from Nayarit are differentiated morphologically. *Phyllodactylus lupitae* (SVL = 64.25) is the largest (mean body size), though very similar in size to *P. cleofasensis* (SVL = 64.19). The smallest species is *P. isabelae* (SVL = 45.91). *Phyllodactylus lupitae* is differentiated from *P. cleofasensis* by a smaller number of dorsal paravertebral tubercles (mean 28.82 versus 47.89). With respect to the continental species compared, we demonstrate that a set of diagnostic characters (THT, TSS, and SLV) allows a clear morphological differentiation of *P. cleofasensis* from the other *Phyllodactylus* species in Mexico (see Table 1 and Diagnosis).

The stomach contents of *P. cleofasensis* coincide with those reported in other studies on *Phyllodactylus* species. We found coleopterans, arachnids, and orthopterans, as well as unidentifiable matter (vegetation, rocks, and insect wings) and shed skin remains. Castro-Franco and Gaviño (1990), on El Coral Island, carried out the only study on the diet of the genus *Phyllodactylus* in insular conditions; they identified coleopterans, hemipterans, hymenopterans, homopterans, orthopterans, and dipterans, as well as larvae of different insects, as part of the diet of *P. lupitae*. In this study, we found Araneae, Coleoptera, and Orthoptera present in for the dietary habits of the gecko population from María Cleofas Island, as well as Nematoda, Lepidoptera, Isoptera, Scorpiones, and Blattodea. Barraza-Soltero (2019) identified the stomach contents from the lizard community on María Cleofas Island, including *Anolis nebulosus*, which consumed almost the same number of prey types as *P. cleofasensis* despite differences in foraging habits. In addition, the identification of shed skin remains in stomach contents from some individuals suggests for the first time keratophagous behavior in insular species from the Central Pacific islands of México.

Finally, a striking result was that we did not find non-native species during explorations on the island, despite María Cleofas Island having suffered ecological disturbances due to anthropogenic activities and the vestiges of man-made constructions visible. To our knowledge, non-native species have been reported on some islands pertaining to Nayarit, specifically the Common gecko *Hemidactylus frenatus* on María Madre Island (Casas-Andreu 1992), Isabel Island (Valdez-Villavicencio and Peralta-García 2008), and El Coral Island (Ramírez-Reyes et al. 2015). Similarly, some non-native species have been reported along the Pacific Coast of Mexico in recent years, such as the Brown anole (*Anolis sagrei*, Pazos-Nava et al. 2019), the Mourning Gecko (*Lepidodactylus lugubris*, Ahumada-Carrillo and Weatherman 2018), and possibly a gecko species of the genus *Gehyra* (IKB-S pers. obs.). These species are human commensals, frequently transported intentionally or unintentionally to new localities. Therefore, it is urgent to establish a protocol of species monitoring on María Cleofas Island to prevent the entry of non-native species, which may be a direct threat to native species and disturb balance of insular ecosystems.

## Supplementary Material

XML Treatment for Phyllodactylus cleofasensis
